# Advances and Trends in Forming Curved Extrusion Profiles

**DOI:** 10.3390/ma14071603

**Published:** 2021-03-25

**Authors:** Wenbin Zhou, Zhutao Shao, Junquan Yu, Jianguo Lin

**Affiliations:** Department of Mechanical Engineering, Imperial College London, London SW7 2AZ, UK; junquan.yu@imperial.ac.uk (J.Y.); jianguo.lin@imperial.ac.uk (J.L.)

**Keywords:** profiles/sections, lightweight, curvature, bending defects, extrusion, differential material flow

## Abstract

Curved profiles/sections have been widely used for manufacturing lightweight structures with high stiffness and strength due to aerodynamics, structural properties, and design reasons. Structural components fabricated using curved aluminum profiles satisfy the increasing demands for products used in many high-technology industries such as aerospace, shipbuilding, high-speed rail train, and automobile, which possess the characteristics of lightweight, high strength/stiffness relative to weight, superior aerodynamics performance, and aesthetics. In this paper, the advances and trends in forming techniques of curved extrusion profiles of metal alloys have been reviewed. The curved profile forming techniques are classified into three major categories: conventional cold bending technique, stress/moment superposed cold bending technique, and extrusion-bending integrated forming technique. Processes for innovative development in the field of forming curved profiles are identified; the extrusion-bending integrated technique which can directly form the billets into curved profiles by one single extrusion operation possesses the full potential for further innovation. Due to the nature of the research to date, much of the work referred to relates to hollow circular and rectangular tube cross-sections.

## 1. Introduction

Due to the world-wide demand for sustainable development, production of space and land vehicles with less fuel/energy consumption and CO_2_ emissions is a key aspect that needs to be solved in the Twenty-First century. Research has shown that, in general, for a vehicle weight decrease of 10%, fuel efficiency can be improved by 6–8% when vehicle performance characteristics are maintained [1]. Therefore, lightweight design strategies are effective ways to contribute to environmentally friendly manufacturing of products with low energy consumption, and the need to develop and manufacture lightweight structural components is apparent.

**Figure 1:**
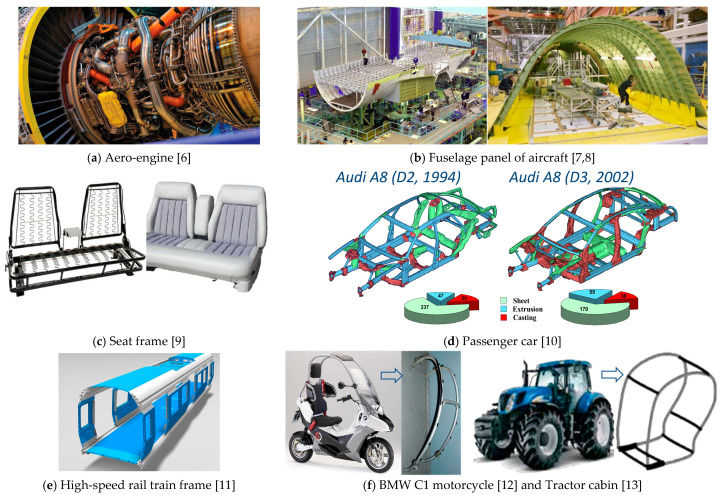
Typical applications of curved profiles in industries.

Lightweight design refers to “the science and the art of making things—parts, products, structures—as light as possible, within constraints” [2]. The application of extrusion profiles/sections for stiff and lightweight design has been widespread. Due to their high strength/stiffness relative to weight, profiles have been used widely for structural applications in designs of all types of vehicles. Profiles used in structures of aircraft, ship, high-speed rail train, and automobile contribute to decreasing the weight of structural components and hence reducing fuel consumption and CO_2_ emissions. For example, lightweight extrusion profiles of aluminum and magnesium alloys with wall thicknesses from 0.25 mm produced by the company MIFA have been used to meet all the requirements set by the aerospace industry [3]. To further achieve superior aerodynamic efficiency, aesthetics and function requirements of structural components, curved profiles with complex curved shapes are used, in contrast to straight profiles commonly used in static structures [4,5,6,7,8,9,10,11,12,13,14,15,16,17,18]. Figure 1 shows typical applications of curved profiles in structural components of industries [6,7,8,9,10,11,12,13].

Curved profiles can be used to meet a large number of structural requirements since they offer a greater freedom of design geometry [4], achievable without resort to the subsequent production steps like cutting, welding/joining [19,20,21], and assembly, necessary with straight profiles. As a result, greatly improved aerodynamic properties for vehicle bodies, increased manufacturing/production efficiency, and decreased production costs of the entire production chain can be achieved [5,22,23]. In addition, to improve aesthetics and expand the range of structural design concepts, curved profiles are extensively used as constructional elements for civil engineering and architectural structures, such as bridges, office furniture, doors, windows, building roof trusses, and building facades/fronts, including sunshades and light shelves [10,14,15,16,17]. Considering the increasing requirement for lightweight structures with decreased aerodynamic resistance and improved aesthetics in the industries such as aerospace, land vehicles, civil engineering, and architecture industry, ready availability of accurately curved profiles with well-defined properties is becoming a vital necessity [24,25,26,27].

In this paper, the advances and trends in forming methods of curved metal profiles are reviewed. The potential defects occurred in manufacturing curved profiles using traditional cold bending methods and the underlying reasons are presented first. To mitigate these defects, the major advances in refining these processes are briefly reviewed, followed by the recently developed stress/moment superposed cold bending technique. Then, the extrusion-bending integrated forming technique, which has been developed as an important way to fundamentally avoid bending defects and increase productivity, is reviewed. Finally, the advantages and disadvantages of curved profile forming methods are discussed and summarized and outlook is given on future development trends.

## 2. Potential Defects Arising in Manufacturing Curved Profiles

**Figure 2:**
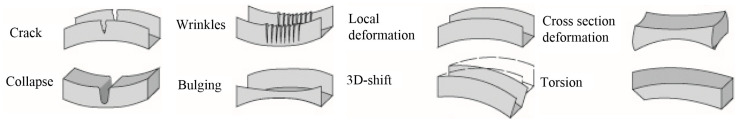
Specific problems for forming curved profiles [5,24,25,27].

Curved profiles are mostly manufactured by a subsequent bending process of the semi-finished straight profiles, which are favorably obtained from billets by conventional extrusion. Similar to the bending of sheet metal, springback occurs in the profile bending process due to the existence of residual stress, which results in geometrical deviation and reduced curvature accuracy. Residual stresses are defined as those stresses that remain in the material after the original cause of the stresses has been removed, which are induced by a variety of mechanisms including inelastic (plastic) deformations in cold forming processes or non-uniform/high temperature gradients/thermal mismatch in hot forming/fabrication processes [28,29,30]. In addition, particular defects occur during the profile bending process, such as cross-sectional deformation, twisting of asymmetric cross-sections, or symmetric profiles along non-principle axes, instability of the profile walls (wrinkling). Additionally, the large strains induced, particularly at the extrados, can degrade metallurgical structure [5,24,25,27]. The mechanical defects are shown in Figure 2.

**Figure 3:**
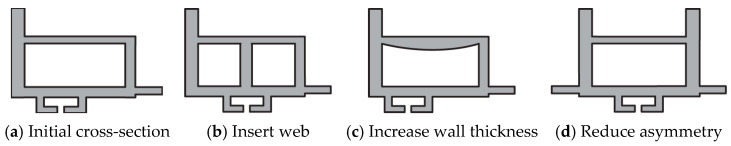
Modifications made to reduce cross-sectional deformation and twisting according to a production-correct design [7].

The cross-sectional deformation of profiles is usually the main problem with profile bending. It becomes increasingly critical with an increase of complexity of the cross-section and tighter geometric specification. Twisting of the cross-section occurs during the bending of asymmetrical profiles where the gravity center (GC) and the shearing center (SC) of the cross-section are not coincident [27]. Modifications according to a production-correct design can be applied to a cross-section in order to reduce cross-sectional deformation and twisting [7], as shown in Figure 3. The most important aspect of these modifications is to increase the profile stiffness and reduce the asymmetry (reduce the distance between GC and SC), which can be realized by addition of webs as well as increase of thickness of walls. Both these actions increase the cross-sectional area and add weight to a section.

## 3. Conventional Process Chain for Manufacturing Curved Profiles and Its Development

**Figure 4:**
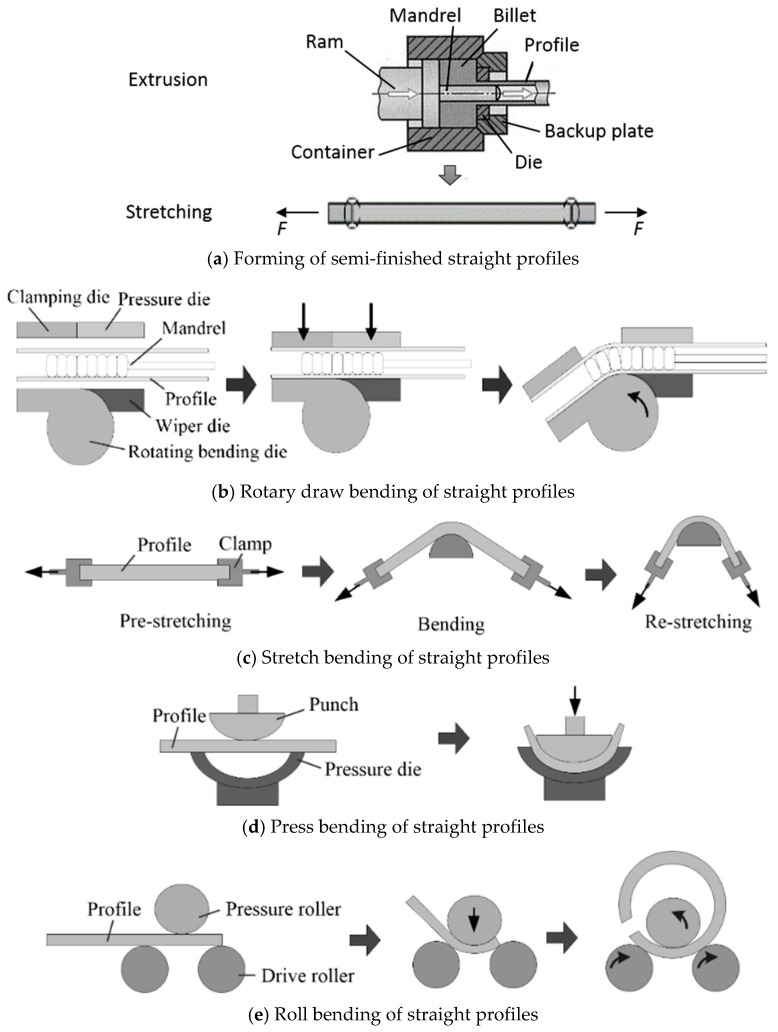
Conventional process chain for manufacturing curved profiles. Reproduced with permission from ref. [27]. Copyright 2014 CIRP.

A conventional process chain for manufacturing curved profiles is shown in Figure 4 [27]. In the first process, billets are extruded into straight profiles with certain cross-sections. Then, stretching is normally needed to make sure the semi-finished profile is straight. Lastly, the semi-finished straight profiles are bent into curved shapes using an external bending device. Various techniques for bending straight profiles have already been employed since the beginning of the twentieth century, which are mostly done by conventional cold bending methods [27]. The most commonly used for profiles are rotary draw bending, stretch bending, press bending, and roll bending, as shown in Figure 4b–e.

### 3.1. Rotary Draw Bending of Profiles

**Figure 5:**
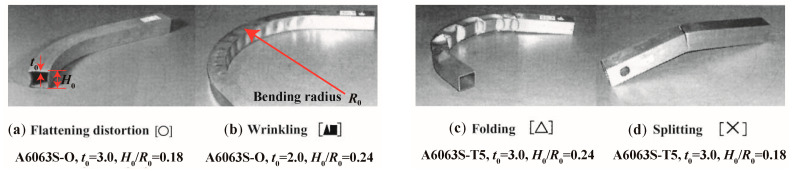
Typical defects occur during rotary draw bending of hollow profiles. Reproduced with permission from ref. [31]. Copyright 2002 Elsevier Science B.V.

Rotary draw bending is a common method used for profile bending. Figure 4b shows the principle of this process. A clamping die holds the profile onto the rotating bending die, while a pressure die is used to support the profile behind the curve and to guide it into the bending area. The pressure die is opposed by a wiper die to provide full lateral constraint of the work-piece. The profile rotates with the die, and it is continuously bent as the die turns. Because of the strict constraint on the profile, rotary draw bending is especially suitable for bending profiles with tight radii. However, due to the high forces needed, undesirable phenomena easily occur in hollow sections, as shown in Figure 5 [31]. Figure 5a shows the flattening distortion that arises on the cross-section when a mandrel is not used. Figure 5b illustrates the wrinkling that occurs on the compression surface when buckling stress is exceeded. Figure 5c shows folding arising when flattening or wrinkling reduces bending rigidity of the section. Figure 5d shows splitting due to high tensile stress on the extrados surface.

**Figure 6:**
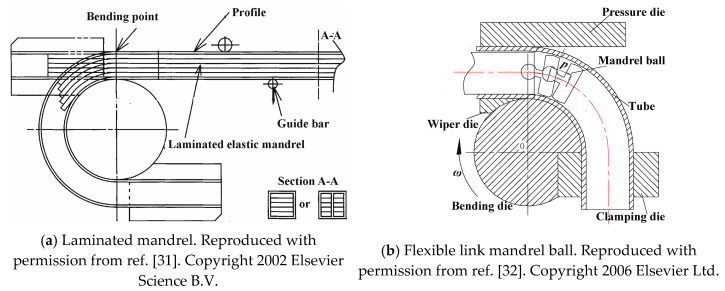
Mandrels used for forming curved profiles [31,32].

**Figure 7:**
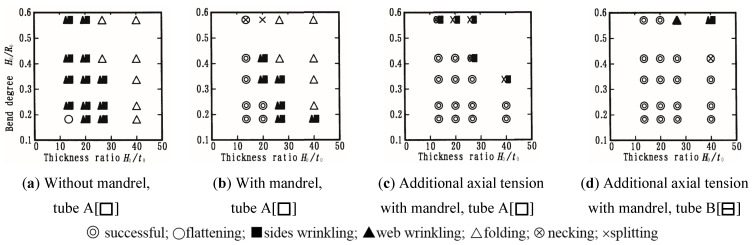
Effects of mandrel and axial tension in the rotary draw bending process. Reproduced with permission from ref. [31]. Copyright 2002 Elsevier Science B.V.

In order to avoid potential cross-sectional distortion, various solid fillers, such as low melting point metals, sand, fluid fillers, and gaseous fillers, can be used prior to bending [25]. These fillers are hard to remove and clean away, which ineluctably increases production cost and environmental pollution. Mandrels which are laminated [31] or articulated [32] have been used to overcome the above problems and decrease cross-sectional deformation. Mandrels including those of thin sheet metal, polymer (Figure 6a) [31,33,34], and flexible link mandrels (Figure 6b) such as articulated mandrel balls [32,35], are inserted into the hollow profile before bending and removed afterwards. A laminated elastic mandrel combined with axial tension was utilized during the rotary draw bending process of aluminum alloy (A6063S-O) square tubes [31,33,34]. The deformation mode diagrams are shown in Figure 7, where parameters *H*_0_, *t*_0_, and *R*_0_ are defined in Figure 5. Flattening distortion can be restrained by the application of a mandrel, and wrinkling and folding can be reduced by applying axial tension. By using a square tube with a center web (type B), splitting can be restrained and the working limit (maximum bend degree) is improved.

An analytical model of the flexible link mandrel balls (Figure 6b) has been developed to preliminarily select the mandrel parameters (mandrel diameter, number of balls, thickness of balls, space length between balls, and nose radius which plays the function of smooth transition) [32]. The effect of mandrel balls has been numerically studied using the finite element (FE) code ABAQUS/Explicit. It was found that increasing the ball number reduces the cross-sectional distortion of the LF2 aluminum alloy tube, but when it exceeds the range preliminarily analytically determined, the role for controlling the distortion is limited and may make the outside tube over-thinning.

**Figure 8:**
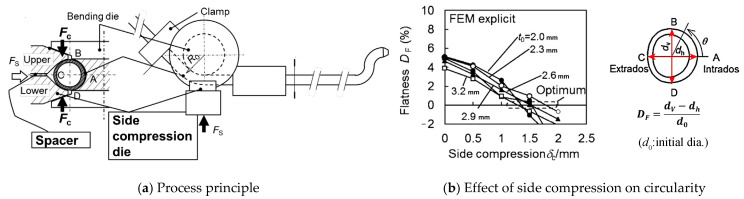
Side compression bending of profiles. Reproduced with permission from ref. [36]. Copyright 2013 CIRP.

Fillers and mandrels inevitably increase tooling costs and dramatically decrease the productivity. Kuboki et al. [36] developed a schedule-free mandrel-less draw-bending procedure, named side compression bending (S.C. bending), as shown in Figure 8a. When the bending die rotates, the side compression dies (upper and lower dies) clamp the tube in the vertical direction (BD) to shrink its vertical diameter and push it towards the rotating bending die, the spacer between the dies regulates the side compression stroke which is the total displacement of the upper and lower dies. For conventional rotary drawing bending with mandrel, the bending process is conducted from the head to the tail in order. S.C. bending is applicable for pre-shaped long tubes and is able to bend tubes in an arbitrary order. The apparatus can be placed at arbitrary positions in a production line, thus offering significant flexibility in manufacturing. Numerical analysis and experiments have been conducted using 5056 aluminum alloys [36]. As shown in Figure 8b, the error index of circularity was evaluated using flatness defined as $D_{F}=\left(d_{V}-d_{h}\right)/d_{0}$, where $d_{V}$ and $d_{h}$ are the vertical and horizontal diameters, respectively. For circular tubes, when the proper side compression δC (indicated as optimum) is adopted, the error index of circularity becomes zero regardless of thickness *t*_0_ (bending radius ratio *R*_0_/*d*_0_ = 3.0).

### 3.2. Stretch Bending of Profiles

Stretch bending is one of the most widely used cold forming method for bending profiles. Figure 4c shows the principle of this process. Bending is achieved by clamping the work-piece at each end and applying an axial tensile force to gradually stretch it over a rounded, fixed bending die. Three basic procedures exist for stretch bending, namely, pre-stretching, bending, and re-stretching [25,37]. The material is fully plastified before or during bending due to a tensile force which stretches the profile in the longitudinal direction, thus leading to a displacement of the neutral layer towards the compression zone and reduced springback [38,39]. However, similar to the rotary draw bending process, mandrels and internal pressure are usually used during stretch bending to support the cross-sectional profile and avoid deformation which occurs in hollow profiles. In addition, both clamped ends of the profiles need to be removed, which can lead to a wastage rate of more than 20%, significantly increasing product cost.

The magnitude of the tensile force has to be properly controlled. When the value of the tensile force is sufficient to move the neutral layer towards the compression zone of the cross-section, increasing the tensile force will not be beneficial for reduction of springback, but it will lead to greater wall thinning and cross-sectional deformation. Paulsen et al. [40,41,42] studied local wrinkling of a rectangular tube in stretch bending, and a design method for prediction of dimensions was proposed. Clausen et al. [43] studied the effects of various parameters on material response in stretch bending of rectangular hollow profiles. They found that the primary parameters influencing local cross-sectional deformation are tensile force level and geometry, and the springback during unloading is mainly controlled by the pre-stretching force level and the strain hardening properties of the material. The selection of the level of stretching or internal pressure for optimum forming is mostly empirical. Miller et al. [44,45] developed a custom bend-stretch-pressure forming facility to minimize springback and distortion of the cross-section. The facility is operated by closed-loop systems, which allow feedback control of the process. A process design strategy was suggested to establish the optimal loading history.

**Figure 9:**
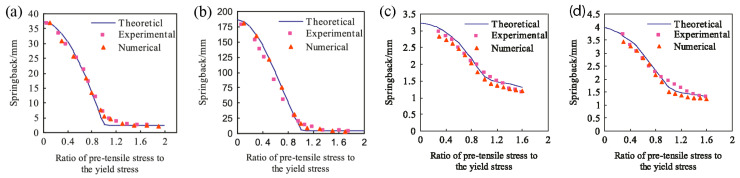
Effect of pretension stress on springback of rectangular section: (**a**) *R* = 150 mm, (**b**) *R* = 300 mm, and U-section: (**c**) *R* = 150 mm, (**d**) *R* = 200 mm, in the stretch bending process. Reproduced with permission from ref. [46]. Copyright 2013 Elsevier Ltd.

A systematic study on plane stretch-bending springback of profiles in the loading method of pretension and moment was carried out [46]. The basic hypothesis that wrinkling is negligible for a relatively large bending radius was made, and springback can be expressed as $\rho_{p}=\left[\rho -\left(\sigma_{T}/E\right)\rho_{\varepsilon }]/[1-\left(M/EI_{v}\right)\rho_{\varepsilon }\right]$, where $\rho_{p}$ is the residual radius of centroidal layer, ρ is the bending radius of centroidal layer under loading, $\rho_{\varepsilon }$ is the curvature radius of strain neutral layer under loading, E is Young’s modulus, $I_{v}$ is the inertia moment of the cross-section, $\sigma_{T}$ is the pre-tensile stress, and M is the bending moment. Stretch bending of ST12 steel profiles with different bending radii *R* is shown in Figure 9 [46]. Springback decreases gradually as pretension stress increases, and when the value of pretension stress reaches that of the yield stress, springback approaches zero and decreases slightly with increase of the pretension stress. For a given pretension stress, smaller bending radius results in lower springback.

### 3.3. Press Bending of Profiles

Press bending is used mainly for sheet metal bending and few publications are available describing its use for solid or tubular profiles. The basic principle is that a work-piece is bent by a punch and die moving together along a common axis, as shown in Figure 4d. The final curved contour of the profile depends on tool shape and springback of the profile. Press bending can form curved profiles with complex shapes by one single operation, which can greatly enhance the production efficiency. However, lateral deformation of the profile often occurs, as the pressure acting on the profile is high. This may be alleviated to some degree by the use of supporting plates. In addition, to reduce deformation, mandrels, fluid fillers, and gaseous fillers are usually adopted for press bending of hollow profiles.

**Figure 10:**
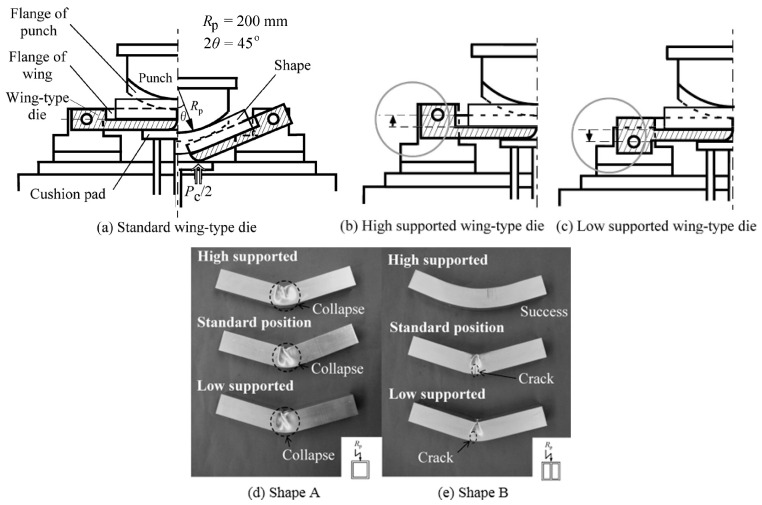
Appearance of shapes after press bending with different support positions of wing-type dies [48].

To eliminate use of a mandrel and simplify the bending operation, a variable die opening system and counterpressure have been utilized to effectively restrain localized deformations [47]. Wing-type dies, which support the sides of a work-piece have been developed [48]. As shown in Figure 10, successful press bending of A6063-T5 alloy shape without collapse was achieved using high supported wing-type dies. This is because the position of wing rotational center affects the sliding direction of the work-piece on the dies. For high supported dies, friction acts to produce axial tension. The tests also revealed that an inner web, parallel to the plane in which the profile is bent, is effective for preventing wrinkling. Similar to other conventional cold bending methods, additional axial tension is helpful to alleviate wrinkling of the compressive side of the profile. The beneficial effect of axial tension in press bending was also studied using AZ31 magnesium alloy square tubes [49]. It was found that under stretch press bending, a tube of AZ31 alloy was successfully bent without wrinkling at room temperature, using axial tension.

**Figure 11:**
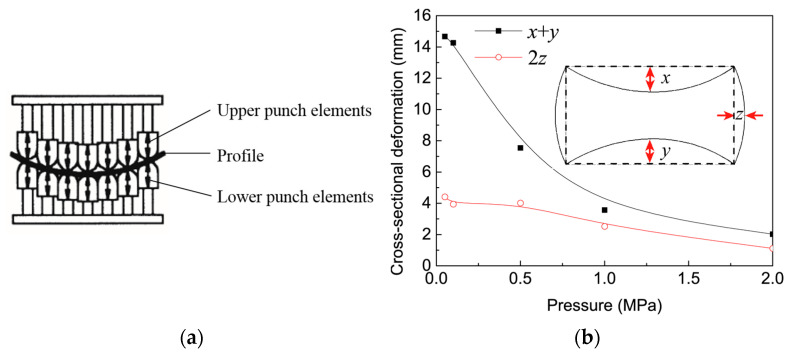
(**a**) Principle of the multi-point bending [50], (**b**) effect of inner pressure on cross-sectional distortion [51].

Multi-point forming (MPF), which was initially developed to form sheet metal parts, has been used to bend hollow A6N01S-T5 profiles [50]. MPF is a flexible manufacturing process with reduced time and costs for die design and fabrication. In MPF, as shown in Figure 11a, a series of discrete punch elements are used to replace the conventional solid dies. The contour shape of the punch matrix can be varied freely as the height of each punch element is independently controlled. By adjusting the relative height of each punch element, the shape contour of the punch matrices is formed to bend the profiles. Figure 11b shows the cross-sectional distortion of an A6N01S-T5 profile (bending radius 2000 mm) in MPF with different levels of inner pressure [51]. It indicates that the inner pressure can effectively restrain the cross-sectional distortion, although the defect is not eliminated.

### 3.4. Roll Bending of Profiles

Roll bending is a relatively economical method for profile bending, commonly using three-, four-, or six rolls. A diagram typifying this process, using which, a minimum bent radius two to three times greater than that achievable by other methods, may be achieved, is shown in Figure 4e. Roll bending is best suited to large production volumes. In three-roll-bending, a work-piece is supported on two level fixed drive rollers and bent by a vertically moving pressure roller positioned above them. The work-piece is bent along its length as it passes through the turning rollers. Therefore, it is possible to vary curvature over the length of a profile by positional control of the pressure roller [52,53]. Compared with three-roll bending, four-roll bending can be used to reduce cross-sectional deformation as the fourth roller supports the lower wall of the profile [54]. S-shaped profiles are made advantageously by six-roll bending, which is essentially mirrored four-roll bending [25]. The main characteristic of roll bending is that material in the deformation zone is mostly elastic; therefore, springback is large. Surface roughening is also notable due to the contact with the rollers. Both ends of rolled profiles cannot be bent and must be cut off, thus leading to material wastage.

**Figure 12:**
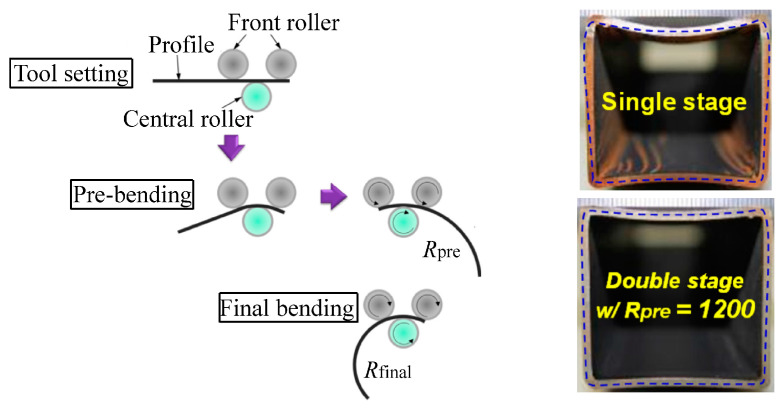
Double-stage forming of profiles with large curvature (pipes: 40 mm × 40 mm × 2 mm). Reproduced with permission from ref. [55]. Copyright 2016 Elsevier B.V.

Double-stage forming using a critical pre-bending radius was proposed to reduce cross-sectional distortion of roll bent hollow profiles [55], as shown in Figure 12. It was assumed that by regulating the pre-bending radius at the pre-bending stage, cross-sectional distortion can be minimized. For a hollow rectangular cross-section of thickness *t* and width *w*, by assuming the initial yielding occurred at the top surface of the midsection, the critical bending radius that represents the beginning of flattening was determined as $R\leq 1.5w^{2}/t$. This expression indicates that the critical bending radius increases with decreasing thickness, i.e., flattening occurs at a small curvature for thin wall thickness. Figure 12 shows the bent STKR490 steel pipes with a cross-section of 40 mm × 40 mm × 2 mm. Compared with single-stage bending, significant improvement on flattening is achieved in the double-stage bending with analytically calculated pre-bending (radius R≤1200 mm). The reduced defect is essentially achieved with a compromise of time or cost, i.e., the deformation caused by bending progresses incrementally and gradually due to smaller forming increments or more forming stages.

**Figure 13:**
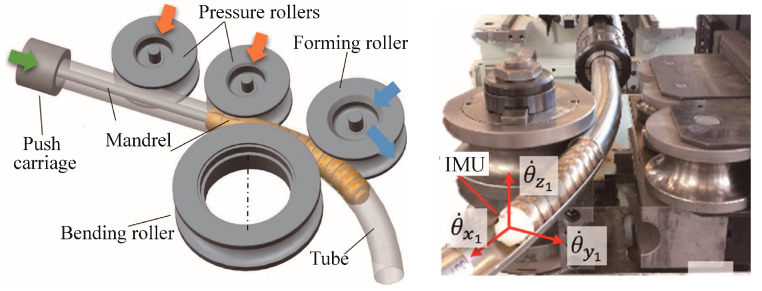
Tools and main kinematics in the three roll push bending (TRPB) process. Reproduced with permission from ref. [56]. Copyright 2017 Published by Elsevier Ltd. on behalf of CIRP.

Three roll push bending (TRPB), shown diagrammatically in Figure 13, has been widely used for manufacturing curved hollow profiles [56]. A push carriage which can rotate around its longitudinal axis is used to grip and move the profile. The profile is forced to pass through one or more pressure rollers, and is shaped incrementally between a bending roller and a forming roller. The pressure rollers support the straight part of the profile to avoid premature deflection and the forming roller, which can translate and rotate with respect to the bending roller, controls the final profile curvature. The TRPB process can be controlled to respond to springback, and an in-line approach for real-time evaluation and correction of the springback of bent profiles during the TRPB process was developed based on inertial measurement techniques [56]. As shown in Figure 13, a novel mandrel, made of individual rings, was developed to monitor profile curvature after springback. The actual bending angle is the actual rotation angle of the last mandrel ring since its orientation is the same as the actual cross-section of the bent profile. A commercially available Inertial Measurement Unit (IMU) is embedded at the last mandrel ring which allows real-time recording of its angular speed around the axes of the profile during the bending process. Being connected to the CNC machine, at each time increment, the springback is calculated to enable a prompt roller adjustment for springback compensation or machine stop. IMU has also been used to detect the onset of wrinkling during the TRPB process.

### 3.5. Springback Prediction Models

**Figure 14:**
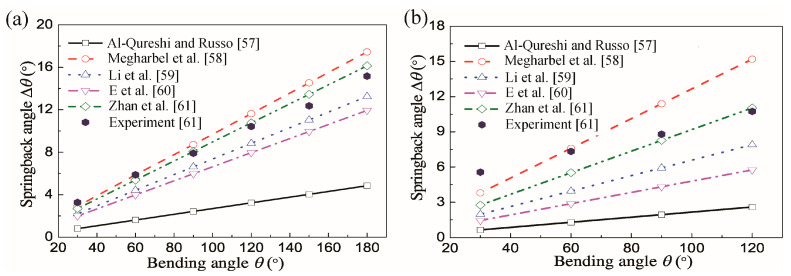
Analytical springback values for Ti-alloy tubes of (**a**) *Ø*6 mm × 0.6 mm (**b**) *Ø*12 mm × 0.9 mm [57,58,59,60,61]. Reproduced with permission from ref. [61]. Copyright 2016 Elsevier B.V.

Elastic recovery (springback) after unloading is an issue that directly affects the dimensional accuracy of bent profiles. Significant springback of profiles occurs after the above-mentioned cold bending processes, especially for materials with high strength and low Young’s modulus, such as for Ti-alloy tubes. Many studies have been conducted on springback analysis using experimental and modelling methods; here, the major models showing differences in predicting springback of Ti-alloy tubes are compared in Table 1 and Figure 14 [57,58,59,60,61].

During the tube bending deformation, the wall thickness and neutral layer usually vary. Zhan et al. [61] found that the Young’s modulus of Ti–3Al–2.5V tubes decreases in the initial stage (after yielding) as function of equivalent strain before stabilizing in the final stage. To examine effects of variations of tube thickness, neutral layer variation/offset and Young’s modulus on springback, an analytical model was established [61]. The springback angle increases nearly linearly with the increase of bending angle. The model of Zhan et al. [61] predicts springback closest to the experimental results. The springback angles predicted by Al-Qureshi and Russo [57] are the lowest since their model considers the tube material to be elastic-perfectly plastic and neglects the hardening effect. By considering tube material to be exponentially work hardening, the model of Megharbel et al. [58] predicts the greatest springback angles. This is because unloading moments are over-estimated by the assumption that they are equal to the bending moments in classic springback theory. The static equilibrium springback theory by Zhan et al. [61] considers the residual moment, thus eliminating over-springback. The similar triangular unloading theory by E et al. [60] utilizes similarity between the unloading triangle and elastic loading triangle to determine unloading stress and strain only through the outer surface stress and strain. It is essentially a method of approximation.

## 4. Stress/Moment Superposed Cold Bending Methods

### 4.1. Compressive or Tensile Stress Superposed Rolling-Bending

**Figure 15:**
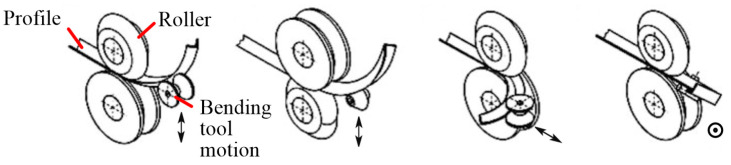
Schematic of the integrated rolling–bending process [38,62]. Reproduced with permission from ref. [38]. Copyright 2007 Springer-Verlag Berlin Heidelberg.

An integrated rolling and bending process, rolling–bending with superposition of compressive stresses, was firstly proposed by Finckenstein et al. [62]. Basic process principles are shown in Figure 15. The section is squeezed to beyond yield between two rolls which have matching profiles and its thickness is reduced. A bending roller situated close behind the squeezing rolls and offset from the roll gap causes the section to bend. Plastification due to squeezing decreases bending resistance and hence bending force. Profile curvature is mainly attributed to superposition of compressive stress rather than bending force. Compared with conventional cold bending, this process enables higher true strains and improved material strength to be achieved. However, this process is restricted to sections formed from sheet or strip and is not applicable to hollow or thick ones.

**Figure 16:**
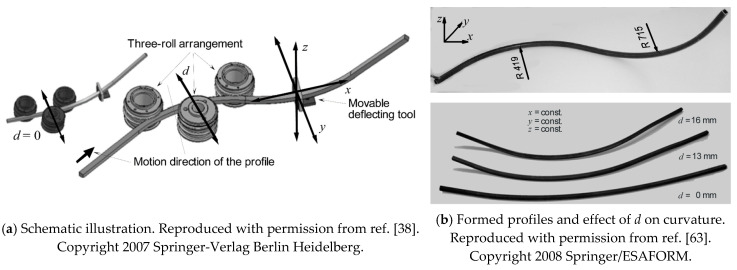
Superposed three-roll-bending with subsequent profile deflection [38,63].

Both stretch bending and integrated rolling–bending incorporate superposition of stress states within the deforming region, tension or compression, respectively, thus facilitating bending. Inspired by the concept of stress superposition, Chatti et al. [38,63] developed a modification of the rolling–bending by superposing three-roll-bending with subsequent profile deflection. The tool configuration for this process is shown in Figure 16a. The two essential stages of this method are; a conventional CNC-three-roll-bending-machine to bend normal to the rolls axes (*xy* plane), and a movable bending tool to deflect the section normal to this plane (*z*-axis) immediately after the previous bending process. As before, initial three-roll-bending creates local pre-plastification in the forming zone of the material, making further bending easier.

A representative formed profile made of S235JR steel is shown in Figure 16b. By selecting different rolling adjustment values (*d* = 0 mm, 13 mm, and 16 mm) for the middle roller, while keeping the bending tool deflection the same (*z* = 40 mm), it was found that an increase of the *d*-adjustment leads to an increase of the profile curvature in the third plane. The increase of the curvature is a result of the decrease of the profile springback due to the stress superposition. It was also found that to achieve the same profile curvature in the third plane, lower bending force is needed for greater *d* due to the increased pre-plastification. The reduced bending force will also decrease the cross-sectional deformation of the profile. However, this method is only applicable for bending profiles with relatively large radii. The friction between the roller and the profile is insufficient for the transportation of the profile when bending profiles with smaller radii.

### 4.2. Torque Superposed Spatial (TSS) Bending

**Figure 17:**
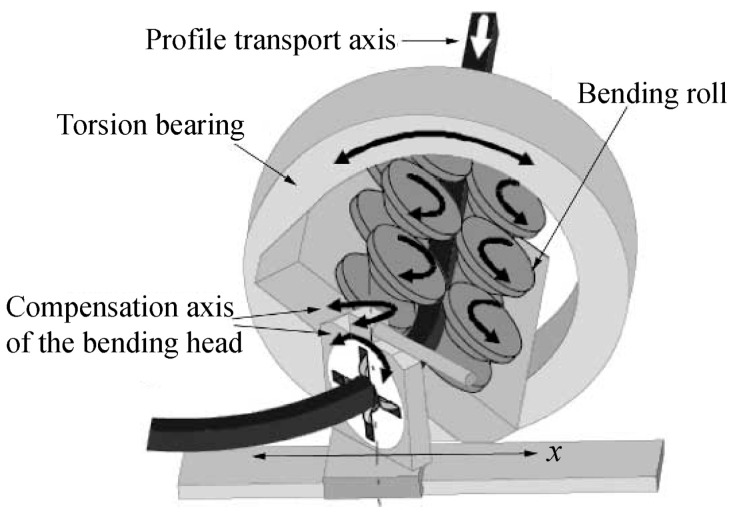
Process of torque superposed spatial (TSS) bending. Reproduced with permission from ref. [65]. Copyright 2010 CIR.

To enable 3D-bending of profiles with smaller radii, Hermes et al. [64,65] developed a 3D profile bending method, torque superposed spatial (TSS) bending, which is shown in Figure 17. It has a six-roll unit (three pairs) to transport and guide the profile over the longitudinal axis, and a guiding system (bending head) composed of four smaller rollers to achieve the bending curve (bending axis *x*) in the horizontal plane. By using the bending head, including bending over axis *x* as well as twisting over its compensation axis, it is possible to bend 2D contours and S-shapes. The 3D-bending is realized by a superposed torque of the three roll pairs over the longitudinal axis.

In the conventional cold bending processes discussed in Section 3, it occurs quite often for asymmetrical profiles to twist over the longitudinal axis since the shearing center and the center of gravity of the cross-section are different. In addition, collision of tool elements occurs especially when profiles with smaller radii are bent. However, during the TSS bending process, twisting of asymmetrical profiles can be compensated by superposition of torque with the bending moment. In addition, the leverarm distance between six-roll unit and bending head can be adjusted, and thus, profiles with a wide range of radii can be bent without tool collision issues. The friction-based six-roll drive enables bending relatively long profiles since no extra pushing system is required. Integration of TSS bending with a continuous section forming process, such as roll-forming, constitutes a facility for continuous production of 3D bent profiles. The prototype machine has been further developed, an induction heating device and a subsequent cooling tool are added, which can heat and then quench the deforming zone and enable the initial process limits to be extended by reducing bending forces. In addition, graded structures over the longitudinal axis can be achieved due to in situ heat treatment [66,67]. The reason is that heat treatment can improve the formability of extruded profiles, thus enhancing the forming limits in bending processes [68,69].

An analytical model with consideration of the elastic deformations of the machine tool and profile during TSS has been developed for springback compensation of S235JR steel profiles [65]. The springback was calculated by the analytical model to determine the compensation value and get the target contour after unloading. Slight contour deviations (less than 3.5 mm) were found in a compensated component, while greater variations (5–50 mm) occurred for the case without considering the elastic deformation.

### 4.3. Incremental Tube Forming (ITF)

**Figure 18:**
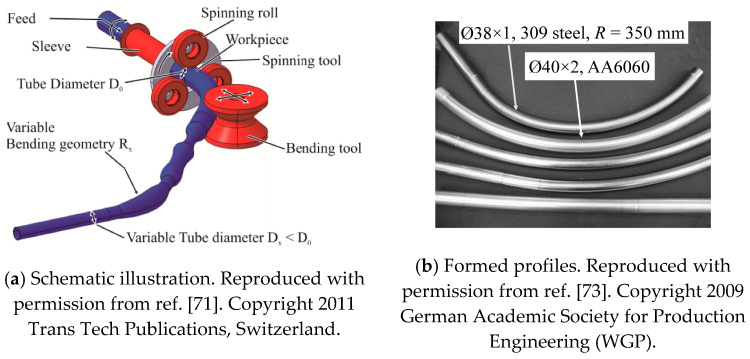
Process of incremental tube forming (ITF) [71,73].

Load-adapted tubes, also called tailored tubes, in which the cross-section changes along their length, are becoming widely used in the automobile industry. Current methods for forming them are very complicated. Hermes et al. [70] developed a method for incrementally forming bent tailored tubes. The basic principle is a combination of a tube spinning and a tube bending, as shown in Figure 18a [71]. A straight tube is drawn through three spinning rollers mounted on a rotating housing which, by moving radially to it, can alter the tube diameter. Subsequently the tube is bent. Additionally, the tube can be rotated to achieve three-dimensionally bent structures. Thanks to the free movability of the spinning rollers and the bending tool, tubes with arbitrary bending radii and diameters along their length may be produced. The ITF process has been further developed by adding a mandrel inside the tube, which significantly extends processing limits [72]. The formed typical profiles are shown in Figure 18b [71,73]. The mandrel prevents folding or cracking of the tube by supporting its inside. Additionally, the mandrel can be utilized to achieve variation of wall thickness of the tube. Precise wall thickness can be achieved by adjusting the gap between spinning rollers and mandrel, and as the tube moves in the axial direction, a continuously variable wall thickness can be realized over the longitudinal axis of the tube.

The benefits of superposing spinning process with bending were studied using AA6060 tubes with a cross-section of *Ø*40 × 2 mm [74]. With the adjustment of the spinning roller as *d* = 0.5 mm, an unloaded bending radius of ~950 mm was achieved. Without superposition of spinning, springback is greater (~1500 mm). The bending force reduction was confirmed using tubes of the same dimension but different materials; the construction steel S235JR, the heat treatable steel 25CrMo4, and the alloyed steel 15CrMoV6 [71]. All tubes were bent to the same radius, 2500 mm. A significant reduction of the bending force was achieved by the spinning process. This is due to the stress superposition of compressive (spinning) and bending stresses. Local plastification of the material, which makes it easier to deform, is generated during the spinning process, and thus, the subsequent superposed bending force, which deflects the tube, is reduced.

## 5. Extrusion-Bending Integrated Forming Methods

As discussed above, conventional cold bending methods and stress superposed cold bending methods for forming curved profiles usually need more than one operation. That is, the first is to form the billets into lengths with prescribed cross-sections (usually by conventional extrusion or shape rolling) which are then cold bent. Two essentially different integrated extrusion-bending methods have been proposed in which the billets are directly extruded into curved profiles/sections; they are reviewed below.

### 5.1. Curved Profile Extrusion (CPE) Using External Bending Apparatus

**Figure 19:**
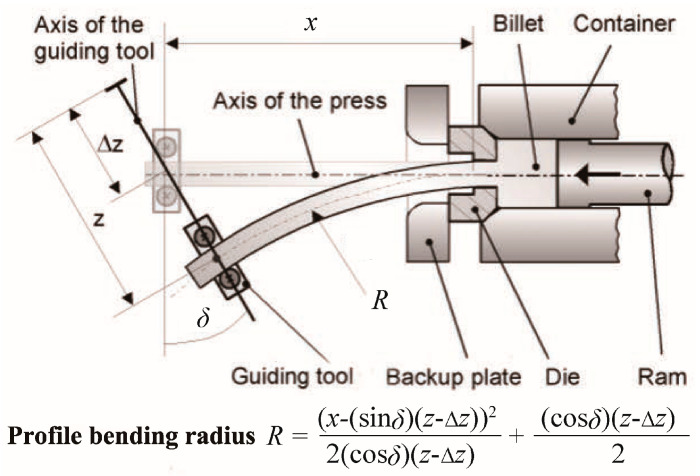
Process principle of curved profile extrusion (CPE) [75,76].

Kleiner et al. [75,76] used an external guiding tool during the extrusion process to form curved profiles, as shown in Figure 19. The contact surface of the guiding tool matches that of the external cross-section of the profile. The predefined path of the tool is controlled by a linear axes system and moves synchronously with the current speed of the extruded profile. The curvature of the profile is generated due to the bending moment applied by the guiding tool. The profile bending radius depends on the geometrical position of the experimental setup, including the distance *x* between the guiding tool and the die, the distance *z* between the position of the guiding tool and the press axis, and the changed angle *δ* for the axis of the guiding tool. The curvature is generated at the die orifice where the material has a high temperature (for hot extrusion) and a relatively low flow stress so that small forces are needed to locate the guiding tool; therefore, this forming process can reduce the cross-sectional distortion and residual stresses [77,78]. It should be noted that press geometry determines minimum radius obtainable since curvature begins within the die orifice, and the profile can collide with the backup plate when the bending radius is small.

**Figure 20:**
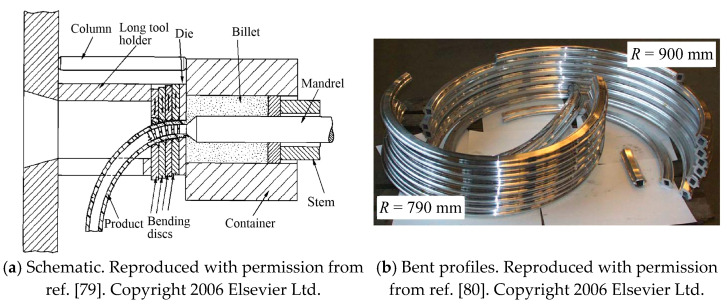
Curved profile extrusion (CPE) using a segmented guiding device [79,80].

The basic process was improved by Müller et al. [79,80] who developed a segmented guiding device using serially placed bending discs, as shown in Figure 20a. Two bending discs are directly placed next to the extrusion die. The disc nearest to the extrusion die defines the location of the start of bending. The second disc can move in two translational directions and also rotate. A die holder is used to hold the bending discs, and a sidewise cut was introduced to the die holder to prevent the outer end of the profile from colliding with the die holder. Curved hollow rectangular aluminum profiles with flanges have been formed using this method (Figure 20b), it was concluded that starting location of the bending curvature should be as close to the bearing surface as possible in order to reduce the remaining stress in the profile caused by the additional hot deformation work during the bending process, and the bending device should also be installed as near as possible to the extrusion die outlet to obtain a small bending radius.

**Figure 21:**
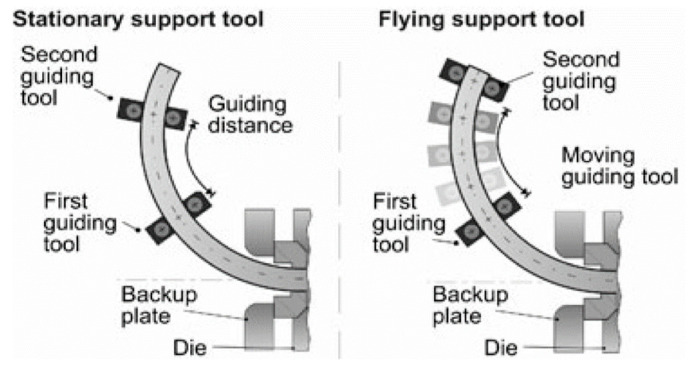
Flexible support for curved profile extrusion (CPE) [12,81,82]. Reproduced with permission from ref. [82]. Copyright 2015 Springer-Verlag Berlin Heidelberg.

Based on Kleiner’s previous research [75,76], since small external bending forces are needed to deflect plastified extrudates, natural forces such as gravity could influence contour accuracy and should be compensated. The effect of profile weight can be compensated for by a run-out table which can provide support for planar bent profiles. Effects of friction between the run-out table and profile can be minimized by using a table surface made of graphite. However, for profiles bent in three dimensions a flat run-out table is ineffective. Klaus et al. [81] developed a second guiding tool for flexible support of profiles. As shown in Figure 21, at the beginning of the CPE process, the second guiding tool is placed at a constant distance behind the first guiding tool, and moves with the profile to maintain its curvature. The effects of this distance and the extrusion speed were studied, which concluded that a long support distance with a slow extrusion velocity results in smaller contour deviations. Becker et al. [12,82] refined the support strategy and introduced flying (moving) guides, between which the distance could be changed (Figure 21). By using the flying support tool, deviations for aluminum profiles could be reduced by 25%. A component-specific scale was further developed by Munzinger et al. [83] to in-line measure the contour, position, and orientation of the extruded curved profiles without having to change the mechanical system.

For heat treatable aluminum alloys, quenching is needed directly after extrusion, to realize fine distribution of alloying elements needed to gain high mechanical properties through subsequent ageing. Becker et al. [12,84] developed an in-line quenching device composed of two cooling rings. The first ring, perforated to expel fine air blasts onto the extrudate, is installed directly behind the die orifice. The second is positioned at the first guiding tool to realize fast cooling such as by spraying a water mist for localized quenching to minimize distortion. Using mist spray cooling in the CPE of aluminum alloy 6060, a cooling rate up to 20 °C/s was achieved. Recently, a flying cutting tool attached to a robot was developed to cut profiles [85,86,87].

### 5.2. Curved Profile Extrusion (CPE) Based on Internal Differential Material Flow

Extrudate curvature can be obtained by inducing non-uniform flow velocity across the die orifice. This can be realized by four different approaches: (i) using an eccentrically positioned mandrel to influence the material flow before the deformation zone, (ii) influencing the material flow after the deformation zone by varying the length and inclination angle of the die bearing/land, (iii) using the multi-orifice die or die with eccentric orifice, and (iv) using an inclined die or a forming pocket before the die bearing/land surface.

Material flow velocity distribution along the profile cross-section before the deformation zone can be modified by utilizing the friction between work-piece and extrusion container. Dajda et al. [88] proposed an approach in a patent for the extrusion of pipe bends and elbows by means of an eccentrically mounted mandrel which results in asymmetric friction between container and billet and thus asymmetric distribution of the material flow velocity before the deformation zone. Curved profiles are thus acquired as a result of this asymmetric velocity distribution.

**Figure 22:**
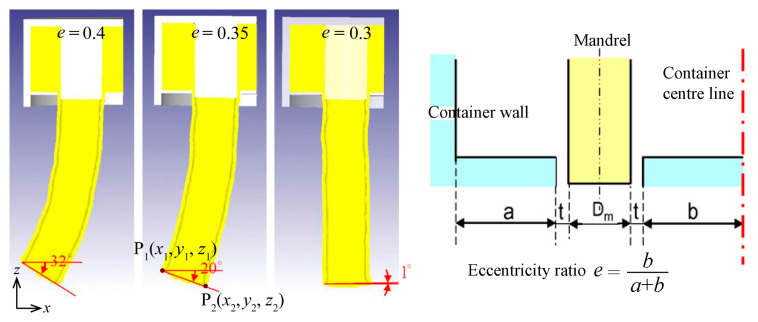
Effect of eccentricity ratio on profile curvature in two-hole extrusion. Reproduced with permission from ref. [89]. Copyright 2007 Elsevier B.V.

A single eccentric die orifice without eccentric mandrel can also result in a curved extrudate, although the distribution of the material flow velocity before the deformation zone remains symmetric. In addition, a multi-orifice die alone can be used to produce curved profiles, even with the same land geometry for every orifice. Degree of curvature depends on the location of an orifice relative to the central axis of the billet. Using multi-orifices can reduce required extrusion force compared with that required for a single orifice. Chen et al. [89] studied the effects of temperature and orifice eccentricity ratio on curvature of aluminum alloy 7075 profiles for the two-hole extrusion process, as shown in Figure 22. It was found that the eccentricity ratio of die orifices has the greatest influence on profile curvature as flow velocity decreases the further is the extrudate from the billet central axis. Lower extrusion temperature results in slightly greater curvature.

**Figure 23:**
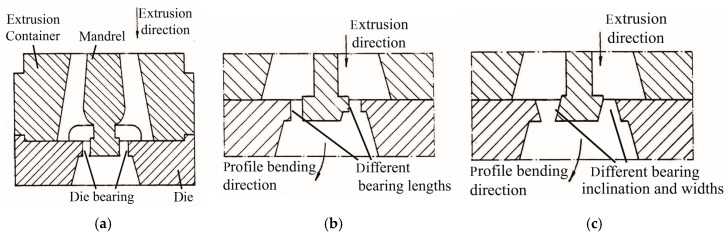
Design of the extrusion die bearing geometry to achieve curved profiles: (**a**) initial equal die bearing, (**b**) different die bearing lengths, (**c**) different inclination angles and gap widths of the die bearing [90].

Distribution of flow velocity along the profile cross-section after work-piece area reduction, can be changed by changing die bearing/land friction through altering bearing/land geometry. This is achieved by either (a) altering bearing/land surface length, or (b) varying inclination angle of the bearing/land, or (c) using different gap widths and inclination angles when bending hollow profiles. Tiekink et al. [90] proposed an apparatus in a patent in which the extrusion gap is bounded by separate, mutually oppositely situated running surfaces, as shown in Figure 23. By varying the shape and/or length of the running surfaces and/or width of the extrusion gap, the material flowing through the extrusion gap experiences a lower resistance at one side of the die than the other side, and a curved profile whose longitudinal axis has a curvature with a certain radius is thus formed, due to the difference in the amount of material at the two sides.

Regulating metal flow by varying the die bearing/land, thus modifying friction, will result in uneven heat being generated over the die bearing/land, which restricts usable extrusion velocity and shortens die life. Additionally, modifying land friction can lead to surface defects in the product and in addition, design of extrusion orifices, especially for thin hollow sections, is challenging and subject to much trial and error modification.

**Figure 24:**
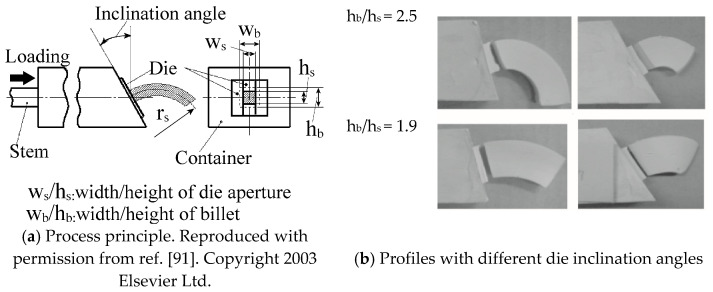
Design of the inclined extrusion die to achieve curved profiles [91,92].

Material flow velocity distribution over a cross-section in the deformation zone can be influenced by exploiting an inclined die. Shiraishi et al. [91,92] proposed an extrusion-bending integrated forming method where a billet is extruded through an inclined die orifice, as illustrated in Figure 24. Curvature of the extruded profiles can be varied by varying the inclination angle, an increase of which leads to increased curvature.

The addition of a pocket (also named recess or pre-chamber or sink-in) in front of the die bearing/land was initially used to produce the continuous long profiles, which can be realized by welding the following billet to the previous billet left in the pocket. This approach makes the extrusion process operate in a semi-continuous way, thus resulting in decreased manufacturing time. It can also be effective approach to control the metal flow during the extrusion process and acquire the required curved profiles [93,94,95,96]. The pocket is used to pre-deform the extrusion billet and control its speed before it passes through the die bearing/land. Die bearing/land length should be as short as possible when designing a proper pocket for a given die. In addition, for the single-bearing die which has one single-bearing length, controlling material flow becomes solely dependent on the design of the pocket, and the variation of the die bearing/land length is neglected, which is beneficial to the permissible extrusion velocity and surface quality of the extrudates.

**Figure 25:**
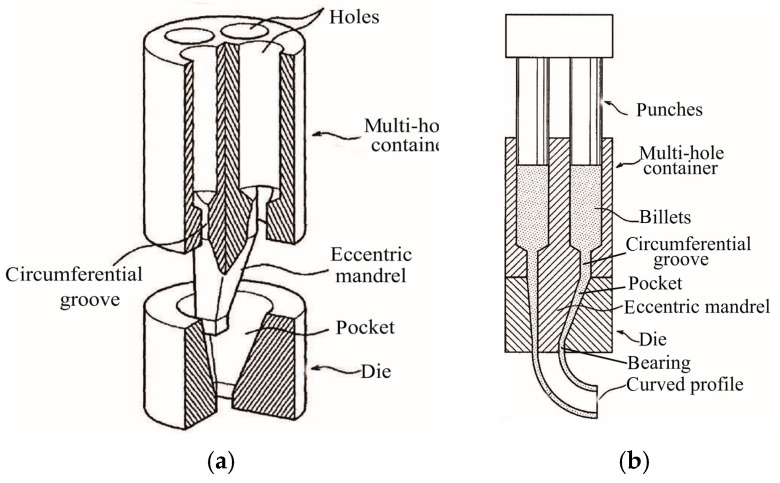
Design of the extrusion die pocket, eccentric mandrel and multi-punch to achieve curved profiles: (**a**) schematic illustration, (**b**) extruded curved profiles [93].

The position, geometry, volume, and step number of the pocket can be designed to achieve control of the velocity of extrusion material flow. Multi-step pocket may also be utilized if the required extrusion pressure is in excess of the press capacity, since the increase in the number of steps in the pocket reduces the peak extrusion pressure required. Jin [93] proposed a device in a patent which is capable of easily and smoothly extruding curved tubes and rods. As shown in Figure 25, four hot metal are inserted into the multi-hole container with an eccentric mandrel. Due to the gradient of extrusion velocities controlled by the eccentricity of the mandrel, or by the relative size of the conical die pocket, or by the relative moving velocity of different punches, curved tubes are obtained.

The pocket die can be used to regulate the material flow easily compared with varying the bearing geometry of the flat die (without pocket), since it can be easily designed and machined. Hence, pocket design is usually preferred, despite utilizing the pocket to control the material flow sometimes having a weaker effect than that of die bearing variation. Pocket die is particularly effective when it is used for manufacturing the multiple-strand extrusions synchronously by a multi-hole die. It becomes more advantageous when it is applied to manufacture a thin-walled profile. However, the pocket design may be combined with bearing length variation to control the material flow when a thin-walled profile has a complex geometry.

### 5.3. Differential Velocity Sideways Extrusion (DVSE) Method

**Figure 26:**
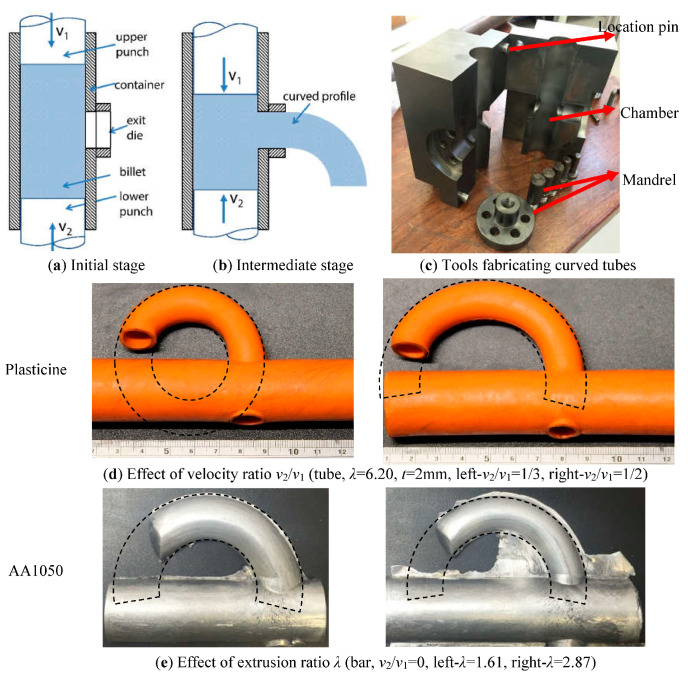
Differential velocity sideways extrusion (DVSE) process for fabricating curved profiles [99,100]. Reproduced with permission from ref. [99]. Copyright 2017 Elsevier Ltd.

It is apparent that profile curvature extruded using the methods described in Section 5.2 has essentially a pre-set constant value. A novel extrusion-bending process termed differential velocity sideways extrusion (DVSE), in which two punches are utilized, was proposed by Zhou et al. [97,98,99], to extrude billets directly into curved sections with adjustable curvatures along the length within one single operation. Figure 26 shows a schematic of DVSE, where the initial situation is shown in Figure 26a, and an intermediate forming stage in Figure 26b. The profile is extruded sideways out of the container and its exiting direction is perpendicular to the punch motion direction. The basic principle of this method is that profiles are extruded and bent simultaneously, due to the gradient of the internal material flow velocity over the die exit orifice caused by the different relative moving velocities of the two extrusion punches. It has been proven by experiments that profile curvature is dependent on the ratio of velocities of the two extrusion punches (0 ≤ *v*_2_/*v*_1_ ≤ 1) and the extrusion ratio *λ*, as shown in Figure 26c–e. Lower velocity ratio (*v*_2_/*v*_1_) and greater extrusion ratio result in greater curvature. For a given extrusion ratio, velocity ratio (*v*_2_/*v*_1_) can be chosen to produce a particular curvature.

**Figure 27:**
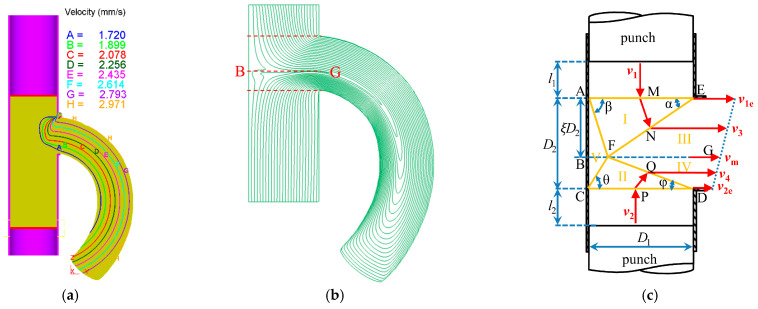
(**a**) Flow velocity gradient across the die orifice, (**b**) flow lines, (**c**) two dimensional deformation model. Reproduced with permission from ref. [100]. Copyright 2018 Elsevier Ltd.

The specific correlation between curvature and velocity ratio and extrusion ratio can be obtained using the analytical upper-bound model and finite element analysis [100,101], which have been widely used in analyzing the extrusion process [102]. As shown in Figure 27a, the extent of the work-piece flow velocity gradient across the die exit orifice, which causes curvature, has been identified. Based on the flow lines shown in Figure 27b, DVSE can be reasonably regarded as two non-equal channel angular pressing (N-ECAP) processes where the dividing line is *BG*. A corresponding two dimensional deformation model considered on diametral planes of container and die is shown in Figure 27c. Above the dividing line, the process of extruding the material in the upper region of the container (diameter $D_{1}$) into the part with the sectional width $\xi D_{2}$ can be regarded as one N-ECAP process. Correspondingly, below the dividing line, another N-ECAP process exists. The eccentricity ratio variable $\xi =g\left(v_{2}/v_{1},\lambda \right)$ represents the effects of $v_{2}/v_{1}$ and λ on the position of line *BG*, which lies in the center of the die exit channel when $v_{2}/v_{1}=1$. As $v_{2}/v_{1}$ decreases, it moves towards the side which has a lower extrusion velocity ($v_{2}$). The theoretically predicted curvature is slightly greater than that from FE modelling and extrusion experiments [100,101]. This is because the effect of die land on flow velocity gradient across the die orifice is not considered due to its small length (2 mm); however, it could have an “unbending” or straightening effect on the extrudate [103], which still needs to be further considered by establishing more sophisticated model.

**Figure 28:**
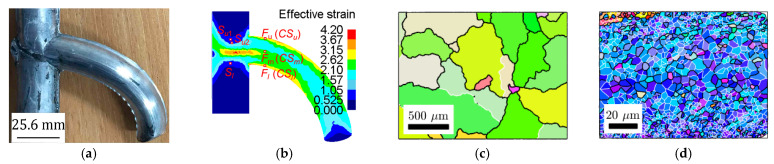
(**a**) Characterized curved profile, (**b**) selected locations for characterization and effective strain, (**c**) microstructure of the billet cross-section, (**d**) microstructure of the profile cross-section. Reproduced with permission from ref. [104]. Copyright 2019 Elsevier Ltd.

Microstructures and mechanical properties of curved profiles formed by DVSE have been examined [104]. The curved extruded profile used for examination is shown in Figure 28a, with a velocity ratio of 1/2 and extrusion ratio of 1.61. When the material in the container passes through the intersection planes, its flow direction changes 90 degree under the action of strong shearing. As a result, severe plastic deformation (SPD) occurs, as can be clearly seen in the simulated effective strains shown in Figure 28b. Selected locations for characterization using electron backscatter diffraction (EBSD) are also shown in Figure 28b. Figure 28c,d show microstructures in the cross-sectional plane of the original billet and formed curved bar (at location *CS_m_*), respectively. The white line indicates the low angle boundaries (LABs, misorientation angle 2° ≤ θ < 15°) and the black line indicates the high angle boundaries (HABs, misorientation angle θ ≥ 15°). Grains of the billet material are large and most of them have HABs, while grains of the curved profile are more equiaxed and homogenous with significantly reduced sizes and increased LABs. The average grain size of the curved profile (~3 μm) is refined to less than 1% of average grain size of the billet (~357 μm). The 0.2% proof stress (yield strength) and ultimate tensile strength of the DVSEed bar have been improved by 354.0% and 116.8%, respectively.

## 6. Discussion

A comparison of the advantages and disadvantages of the profile bending methods is given in Table 2.

Conventional cold bending methods including rotary draw bending, stretch bending, press bending, and roll bending have been widely used for bending profiles with different materials, sizes, and shapes. These bending methods have different characteristics with regard to bending flexibility, profile quality and curvature accuracy. Among them, rotary draw bending and three roll push bending are the most efficient for bending hollow profiles thanks to the availability of advanced CNC machines. Of the two, the former enables higher bending accuracy, since contact areas with forming dies are larger and more severe plastic deformation can be applied, accurate and repeatable shapes with curvature radii from 0.5 to 10 times the diameter of the tube can be obtained with rotary drawing bending. The latter has more flexibility to achieve various shapes and curvature radii with the same tool set, but springback is greater since shape control depends on rollers kinematics and such bending is suitable for curvature radii greater than 10 times the tube diameter. In most practice, work-pieces for forming curved profiles by conventional cold bending methods are previously extruded or roll-formed straight profiles. Bending processes are subject to several undesirable phenomena, such as cross-sectional deformation, wrinkling, and springback, which are also the main scientific problems that the development and optimization in these processes try to solve. Various tools have been developed to avoid bending defects and improve the process capability, e.g., mandrels are employed to avoid cross-sectional deformation and additional axial tension is used to alleviate wrinkling. Additionally, extra compressive stress/moment has been superposed in these cold bending methods, including compressive stress superposed rolling-bending, torque superposed spatial (TSS) bending, and incremental tube forming (ITF). They can reduce the springback, cross-sectional deformation, and wrinkling by reducing the bending force needed. However, essentially these defects are hard to be completely eliminated. Moreover, they inevitably decrease the manufacturing efficiency and increase the production costs.

Extrusion-bending integrated forming methods generally have improved process efficiency, in which the billets are directly extruded into curved profiles/sections. For curved profile extrusion (CPE) using an external bending apparatus, in order to obtain high accuracy of curved profiles, besides the precise control of the guiding tool, all kinematic systems, such as the guiding, supporting and cutting in the CPE process chain, need to be synchronized with the profile extrusion speed. The synchronisation of the guiding tool with the profile speed in the known experiments is realized by synchronizing it with the punch speed considering the press ratio, which might differ from the actual profile speed because of the force-dependent elongation of the press frame (mandrel) and the unsteady temperature conditions. Since the accuracy of each segment depends on the former one, deviations could accumulate towards the end of the complete profile. For CPE based on internal differential material flow, it is quite challenging to achieve high accuracy of curved profiles by controlling the internal metal flow with an eccentric positioned mandrel, forming pocket and inclined die, or by varying the length of the bearing/land surface. Most of them are still in the stage of theoretical design. A lot of trials of die tool design have to be done to get the desired curvature. In addition, since the curvature is not easy to be varied during the forming process, different tool design is needed for profiles with variable curvatures.

The internal differential material flow which is caused by die design (eccentric mandrel/hole, multi-hole die, die bearing/land length or inclination, die pocket, etc.) is fixed or hard to be adjusted during the extrusion process. DVSE is a novel process based on internal differential material flow caused by external differential extrusion velocity ratios rather than die design. It can be used for forming complex-shaped profiles with adjustable curvatures along the length in one extrusion-bending procedure [105], which greatly increases the manufacturing efficiency. It also has the following advantages: (i) forming curved profiles without defects such as distortion or thinning of the cross-section, wrinkling, and folding. Bending is intrinsic to the process, based on internal differential material flow, rather than external bending force; (ii) forming profiles with fine grain size and therefore improved mechanical properties due to severe plastic deformation (SPD) caused by shear stresses in the intersecting deformation zone of the container; and (iii) no fillers or extra heavy machines are needed for the bending process. However, it also has disadvantages, such as a high demand for the specialized extrusion device (double-action or multi-action), and tool design especially for extruding hollow profiles where a mandrel is used, since the mandrel needs to suffer a quite large lateral force due to the profile exiting direction being perpendicular to the punch movement direction.

Diagnosis or prediction of bending defects is of vital importance, considering that some of them are inevitable, e.g., springback for cold bending. Bending defects are influenced by not only the formed profile, cross-sectional geometry, size, and alloy properties, but also process boundary conditions which vary from case to case. Normally, potential bending defects should be diagnosed or evaluated in process design or the first practical trial, to properly tune process parameters for its compensation. The developed techniques for bending defects diagnosis/evaluation include off-line optimization approaches and in-line measurements. Off-line approaches including numerical (finite element) and analytical models are utilized at the first stage of process design. They have the advantage of optimizing process parameters for minimum cost, especially for complex cases using numerical modelling. However, a disadvantage of numerical modelling is long simulation times, making it impracticable to consider scatter of material properties that usually occurs in batch production. Generalized FE software packages such as Abaqus and Ansys are extensively used to model the defects during cold bending processes; however, they require a relatively longer computation time, especially for large deformations. Specialized software products such as Deform and QForm have been widely used for analyzing bulk metal forming processes involving large deformations, they provide good convergence by auto-remeshing and relatively lower computation time. In Deform, the rigid-plastic object is usually assumed; however, to simulate the springback caused by elastic recovery, an elasto-plastic object needs to be used which requires more solution time. QForm is a more efficient software for bulk metal forming processes such as extrusion and forging and needs relatively lower computation time than Deform. Analytical models are restricted to profiles with simple cross-sections and assumptions about the profile, material behavior and the neutral layer, have to be made to get the mathematical solutions.

Close-loop in-line approaches can be used to monitor and control the bending processes. To in-line tune the process parameters, it is necessary to measure the profile continuously and provide rapid feedback to the machine controllers. For example, a feasible method is to use mandrels with embedded sensors. A sensor can be embedded in the mandrel last ring to record its orientation during all the stages of the production process. The sensor data can be analyzed to monitor in real time the actual bending radius in a three roll push bending operation, the springback angle in a rotary draw bending operation, and the onset of wrinkles in both processes. Alternatively, a set of sensors can be positioned on most of the elements of the bending tooling, such as the bending die, wiper die, and mandrel for the rotary draw bending, to dynamically diagnose the bending defects [106]. To achieve this, the hardware and software acquisition system, the sensor calibration procedure and the analysis procedure of both the machine and sensor data need to be developed as well.

## 7. Conclusions and Future Trends in Research and Development

In this paper, the advances and trends on fabricating curved lightweight profiles have been reviewed. Current state-of-the-art shows:

Although conventional cold bending techniques have been well developed and widely applied, most of them cause defects in profiles such as cross-sectional deformation, wrinkling, and springback. Costly tooling is needed to control and mitigate the defects, which decreases manufacturing efficiency and increases production cost. Some modified methods have been proposed based on these conventional cold bending techniques for improving their capability. Since they are still the most developed and extensively used profile bending techniques in industries, the effects of the processing parameters on the bending defects and curvature accuracy should be investigated further to develop the cold bending techniques.

Stress/moment superposed cold bending techniques can realize bending profiles with less cross-sectional deformations, springback and other defects, thanks to the superposition of stress or torsion with the external bending moment. The superposed stress results in local pre-plastification of the material in the forming zone reduces the bending moment/force needed in the subsequent bending process. Twisting of asymmetrical profiles can be compensated due to the superposition of torque with bending moment in the subsequent bending process. However, similarly to conventional cold bending techniques, they normally start with manufacturing straight profiles with predefined cross-sections by shape rolling or extrusion of billets, then the semi-finished profiles are cold bent. Therefore, their manufacturing productivity of profiles is largely reduced since more than one procedure is needed. Nevertheless they still possess the potential to be widely industrialized due to improved bending defects compared with conventional bending techniques, and further research needs to be carried out, to generate sufficient data for process parameters optimization and increase of production efficiency.

Extrusion-bending integrated forming techniques can directly form the metal billets into curved profiles by only one extrusion operation, thus greatly improving the manufacturing productivity. There are basically two principles, either using an external kinematic bending apparatus to influence material flow at the die exit orifice during the extrusion process, or introducing internal asymmetry in the material flow through tool design. Since the curvature is generated at the die orifice where the material is still in the fully plastic state, this forming process produces profiles with reduced residual stresses, springback, and minimal cross-sectional deformations. Therefore, they are quite promising to be widely applied in industries, although they need to be more thoroughly studied for further development. For curved profile extrusion (CPE) using an external bending apparatus, developing more appropriate precise control strategy as well as online measurement systems to improve profile contour accuracy is necessary and yet to be developed. For CPE based on internal differential material flow, new forming processes, with more flexibilities for curvature control without changing the tool design, and abilities to improve mechanical properties of formed curved profiles, are of significant importance.

## Figures and Tables

**Table 1:** Comparison of the springback prediction models.

Springback Model	Material Model	Principle of Springback	Basic Assumptions
2002 Al-Qureshi et al. [57]	Elastic-perfectly plastic	Classical springback theory	Strain-hardening is neglected; Plane strain condition; Cross-section remains symmetrical; Wall thinning is neglected; Variation in neutral layer is neglected
2008 Megharbel et al. [58]	Elastic-exponent hardening plastic	Classical springback theory	Plane strain condition; Cross-section remains in a plane; Wall thinning is neglected; Variation in neutral layer is neglected; Unloading moments equals to bending moments
2012 Li et al. [59]	Exponent hardening plastic	Classical springback theory	Plane strain condition; Cross-section remains in a plane; Wall thinning is neglected; Elastic deformation is neglected
2009 E et al. [60]	Exponent hardening plastic	Similarity in unloading triangle to elastic loading triangle	Cross-section remains flat; Stress neutral layer is unchanged; Wall thinning is not considered; Triangle similarity of the tangential deformation during bending/unloading
2016 Zhan et al. [61]	Exponent hardening plastic	Static equilibrium springback theory	Cross-section remains in a plane; Young’s modulus varies with equivalent strain; Stress neutral layer coincides with the strain neutral layer (offset); Flattening is neglected; Inside radius of the tube is constant Unloading moments equals to residual moments

**Table 2:** Comparison of the curved profile forming methods.

Methods	Advantages	Potential Problems
Rotary draw bending	Small bending radii	Heavy-duty machines are required to exert high bending force; Cross-sectional deformation occurs easily; Fillers are needed to bend hollow profiles
Stretch bending	Reduced springback	Clamping ends of profiles need to be removed (High wastage rate); Pre-stretching and re-stretching forces need to be properly controlled
Press bending	Reduced operation; Relatively low tooling costs	Lateral deformation occurs easily; Fillers are needed to bend hollow profiles
Roll bending	Relatively low tooling costs; Varied curvatures over length	Restriction of small radii; Large springback; Profile ends need to be removed (High wastage rate)
Stress superposed rolling-bending	Reduced cross-sectional deformations and springback	Restriction of small radii;
Torque superposed spatial (TSS) bending	Wide range of bending radii and profile cross-sections; Reduced twisting of asymmetrical profiles	For small radii, an extra axis is needed to enable adjustment of the leverarm distance between the six-roll unit and bending head and avoid the collision of tool elements
Incremental tube forming (ITF)	Bending tailored tubes in one procedure; Reduced springback	Can only handle circular tubes; Mandrel is needed to avoid folding/cracking
Curved profile extrusion (CPE) based on external bending apparatus	No springback for hot extrusion; Adjustable curvatures in one extrusion-bending procedure; No extra heavy-duty machines are needed for the bending process	Complex control of the guiding tool; All kinematic systems have to be synchronized with the current profile speed which may differ slightly from the pre-defined profile speed; Restriction of small radii
CPE based on internal differential material flow	No cross-sectional deformations; No springback; No extra heavy-duty machines are needed for the bending process	Complex design of the die tool; Curvature is pre-set or hard to be adjusted
Differential velocity sideways extrusion (DVSE)	No cross-sectional deformations; No springback; Improved mechanical properties due to severe plastic deformation (SPD); Adjustable curvatures in one extrusion-bending procedure; Enhanced production efficiency	Need specialized extrusion equipment (double-action or multi-action); If two or more individual extrusion machines are used, speed control could be complex.

## Data Availability

Data sharing is not applicable to this article.
